# The MuSK-BMP pathway maintains myofiber size in slow muscle through regulation of Akt-mTOR signaling

**DOI:** 10.1186/s13395-023-00329-9

**Published:** 2024-01-03

**Authors:** Diego Jaime, Lauren A. Fish, Laura A. Madigan, Chengjie Xi, Giorgia Piccoli, Madison D. Ewing, Bert Blaauw, Justin R. Fallon

**Affiliations:** 1 Department of Molecular Biology, Cell Biology, and Biochemistry, Brown University, Providence, RI USA; 2 Department of Neuroscience, Brown University, Providence, RI 02912 USA; 3 Veneto Institute of Molecular Medicine (VIMM), Padua, Italy; 4 Department of Biomedical Sciences, University of Padua, Padua, Italy; 5 Carney Institute for Neuroscience, Brown University, Providence, RI USA

**Keywords:** MuSK, Atrophy, Cachexia, mTOR, BMP, IGF1, Slow muscle, Muscle fiber types

## Abstract

Myofiber size regulation is critical in health, disease, and aging. MuSK (muscle-specific kinase) is a BMP (bone morphogenetic protein) co-receptor that promotes and shapes BMP signaling. MuSK is expressed at all neuromuscular junctions and is also present extrasynaptically in the mouse soleus, whose predominantly oxidative fiber composition is akin to that of human muscle. To investigate the role of the MuSK-BMP pathway in vivo, we generated mice lacking the BMP-binding MuSK Ig3 domain. These ∆Ig3-MuSK mice are viable and fertile with innervation levels comparable to wild type. In 3-month-old mice, myofibers are smaller in the slow soleus, but not in the fast tibialis anterior (TA). Transcriptomic analysis revealed soleus-selective decreases in RNA metabolism and protein synthesis pathways as well as dysregulation of IGF1-Akt-mTOR pathway components. Biochemical analysis showed that Akt-mTOR signaling is reduced in soleus but not TA. We propose that the MuSK-BMP pathway acts extrasynaptically to maintain myofiber size in slow muscle by promoting protein synthetic pathways including IGF1-Akt-mTOR signaling. These results reveal a novel mechanism for regulating myofiber size in slow muscle and introduce the MuSK-BMP pathway as a target for promoting muscle growth and combatting atrophy.

**Supplementary Information:**

The online version contains supplementary material available at 10.1186/s13395-023-00329-9.

## Background

Maintaining myofiber size is essential for proper muscle function. Muscle atrophy characterizes aging, disuse, cancer cachexia, and disease [1–3]. Notably, individual muscles and myofiber types are differentially affected in many of these settings [4]. For example, in humans, the soleus muscle is largely spared from sarcopenia, while other lower limb muscles are reduced in size [5]. Moreover, fast myofibers are selectively vulnerable to atrophy in aged humans [6, 7]. In Duchenne muscular dystrophy, limb-girdle muscles are affected in the first years of life, while upper limb muscles are spared until several years later [8]. In contrast, muscle weakness preferentially affects muscles in the anterior compartments of the face and leg in FSHD [4]. However, the molecular basis for such muscle-selective vulnerability to atrophy is largely unknown.

Members of the TGF-β (transforming growth factor-beta) superfamily, including myostatin and BMPs, are potent regulators of muscle size. Myostatin is a negative regulator of muscle mass and its genetic deletion or pharmacological inhibition results in muscle hypertrophy [9]. In contrast, BMP signaling promotes muscle growth. Overexpression of BMP7 or a constitutively active BMP receptor BMPR1a (ALK3) in skeletal muscle results in increased muscle mass, fiber size, and elevated canonical BMP and Akt/mTOR signaling [10]. Inhibiting BMP signaling by overexpressing the BMP sequestering protein noggin abolishes the hypertrophic phenotype observed in myostatin-deficient mice [11]. Either increasing BMP or reducing myostatin signaling can restore myofiber size in cancer cachexia models [2, 12]. These studies suggest that the BMP and myostatin/activin pathways antagonize each other, and tipping the balance can result in either hypertrophy or atrophy. They also implicate BMP signaling as an attractive target for combatting atrophy. However, targeting BMPs therapeutically is challenging since unlike myostatin, BMPs are expressed ubiquitously and serve a wide range of functions throughout the body [13, 14].

We recently discovered that MuSK (muscle-specific kinase) is a BMP co-receptor that promotes and shapes the BMP-induced transcriptional output in myogenic cells [15]. MuSK binds BMPs 2, 4, and 7 with low nanomolar affinity as well as the type I BMP receptors BMPR1a and 1b. Importantly, the MuSK Ig3 domain is necessary for high-affinity BMP binding. Studies in cell culture show that MuSK enhances BMP4-induced pSmad1/5/8 signaling, and that the expression of a large subset of BMP4-responsive transcripts is MuSK dependent. Notably, this regulation requires the activity of type I BMP receptors, but not that of the MuSK tyrosine kinase. We term this signaling mechanism the MuSK-BMP pathway. The function of MuSK as a BMP co-receptor is structurally and functionally distinct from its role in agrin-LRP4 signaling, which is essential for synapse formation, is mediated by the MuSK Ig1 domain, and absolutely requires MuSK tyrosine kinase activity [16, 17].

The function of the MuSK-BMP pathway in regulating myofiber size in vivo is unknown. However, several observations suggest that this pathway may be particularly important in slow (e.g., soleus) as compared to fast (e.g., EDL and TA) muscles. For example, while MuSK is localized at all NMJs, it is also present extrasynaptically in soleus, but not TA [18]. Further, single nuclei sequencing has shown that MuSK transcripts are expressed at elevated levels in soleus as compared to TA myonuclei, which is consistent with the 3– to 5-fold higher levels of MuSK transcripts in soleus as compared to fast muscle [15, 18].

Here, we investigated the role of the MuSK-BMP pathway in fast and slow muscle in 3-month-old mice. To selectively manipulate the MuSK-BMP pathway, we took advantage of previous work showing that the MuSK Ig3 domain is necessary for high-affinity BMP binding [15] but dispensable for agrin-LRP4 binding and AChR clustering [19–21]. We generated mice where the MuSK Ig3 domain was deleted (“∆Ig3-MuSK”). These mice are fertile and viable with normal innervation levels. Cultured ∆Ig3-MuSK myoblasts show reduced BMP signaling as judged by BMP-induced pSmad1/5 and gene expression. In vivo, ΔIg3-MuSK soleus muscle fibers were reduced in size, while TA fibers were unaffected. RNA-seq revealed largely nonoverlapping changes in transcriptomic profiles and biological GO pathways as well as dysregulation of IGF1-Akt-mTOR components in ∆Ig3-MuSK soleus compared to TA. Notably, in soleus, multiple GO pathways involving RNA metabolism were selectively downregulated. Biochemical analysis revealed soleus-selective reduction of p4EBP1 and P-S6, key mediators of the mTOR pathway. The soleus also showed extracellular matrix (ECM) remodeling including increased deposition of type I collagen. We propose that the MuSK-BMP pathway promotes protein synthesis and maintains myofiber size in slow muscle via the Akt-mTOR pathway.

## Results

### Generation of ∆Ig3-MuSK mice

**Fig. 1:**
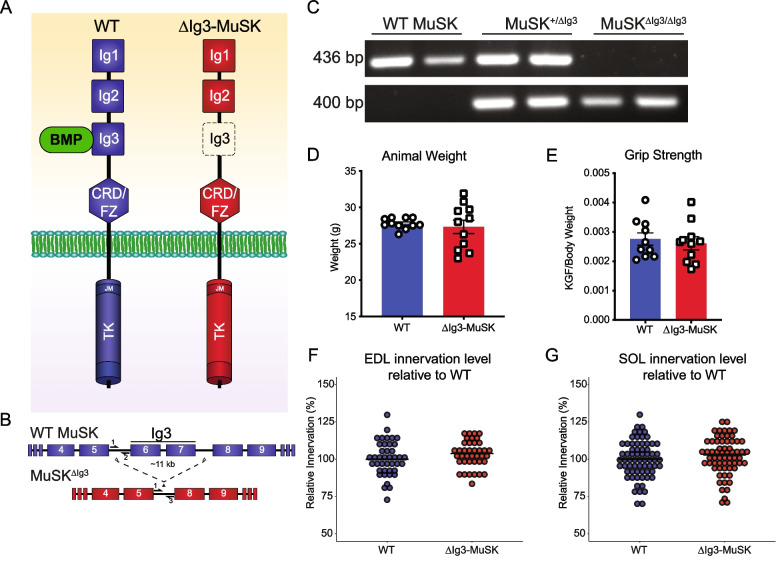
Generation of the ∆Ig3-MuSK mouse. **A** Schematic representation of full length and ∆Ig3-MuSK isoforms. The extracellular full-length isoform contains three immunoglobulin (Ig)-like domains (Ig1, Ig2, and Ig3) as well as a CRD/Fz and an intracellular tyrosine kinase domain. ∆Ig3-MuSK lacks the BMP-binding Ig3 domain. **B** CRISPR-Cas9 was used to delete exon 6 and 7, which encode the Ig3 domain, along with the intervening intronic sequence to generate the ∆Ig3-MuSK allele. **C** PCR amplification of WT and ∆Ig3-MuSK alleles in WT and ∆Ig3-MuSK heterozygous and homozygous mice using allele-specific primers (depicted in **B**). The animal weights (**D**) and grip strength (**E**) of 3-month-old WT and ∆Ig3-MuSK were comparable. Innervation of Ig3-MuSK NMJs, as assessed by overlap of pre- and postsynaptic structures normalized to WT overlap, was equivalent to that in WT in both the fast EDL (**F**) and slow soleus (**G**) (EDL: *n* = 3 animals per genotype, SOL: *n* = 6 animals per genotype, *t*-test n.s. in both muscles)

The MuSK Ig3 domain is necessary for high-affinity BMP4 binding but is dispensable for agrin-LRP4 binding and AChR clustering [15, 16, 20]. To selectively perturb the MuSK-BMP pathway, we generated a mouse model lacking the MuSK Ig3 domain (∆Ig3-MuSK). We used CRISPR-Cas9 to delete an ~ 11-kb genomic region containing exons 6 and 7, which encode the MuSK Ig3 domain, to create the MuSK^∆Ig3^ allele (Fig. 1A, B). PCR amplification of the intronic regions of exon 5–6 and 5–8 borders yielded amplicons of the predicted size for the WT MuSK and MuSK^∆Ig3^ alleles, respectively (Fig. 1C). MuSK^∆Ig3/∆Ig3^ mice are viable and fertile with normal weights (Fig. 1D), grip strength (Fig. 1E), and innervation levels in both the fast sternomastoid (Fig. 1F) and the slow soleus (Fig. 1G). In this study, we will term the MuSK^∆Ig3/∆Ig3^ animals “∆Ig3-MuSK.”

### BMP4 signaling is perturbed in ∆Ig3-MuSK cells

**Fig. 2:**
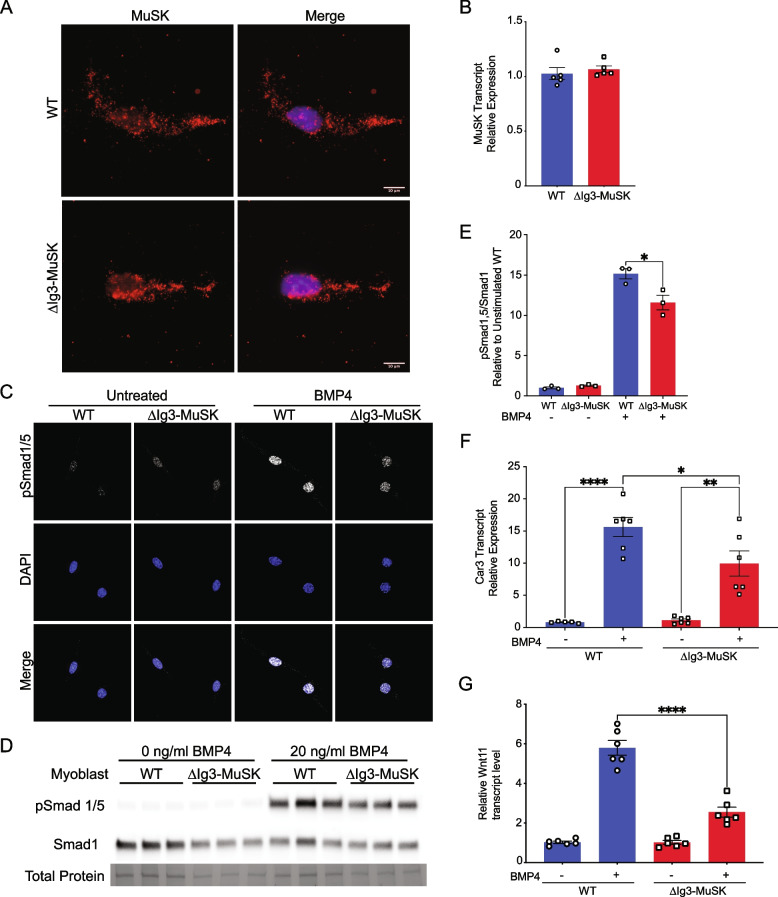
BMP signaling is selectively perturbed in ∆Ig3-MuSK myogenic cells. **A** and **B** MuSK expression: **A** Unpermeabilized WT and ∆Ig3-MuSK myoblasts cells were fixed, and cell surface MuSK was visualized by immunostaining with an antibody directed against the MuSK Ig2 domain (red). Note that comparable levels and distribution of immunostaining were observed in both genotypes. **B** WT and ∆Ig3-MuSK myoblast cell lines express comparable levels of MuSK transcript as assessed by qRT-PCR. Data are means ± SEM from five biological replicates and two independent experiments. **C**–**G** BMP signaling: **C** WT and ∆Ig3-MuSK myoblasts were treated with 20 ng/ml BMP4 for 15 min and immunostained for pSmad1/5. Note the increased intensity of pSmad staining in nuclei of BMP4-treated WT compared to ∆Ig3-MuSK cells. Data are from three independent experiments with three biological replicates per group. **D** pSmad1/5 and total Smad1 in BMP4-treated cells (as in **C**) were assessed by Western blotting. **E** The level of BMP4-induced pSmad1/5, normalized to total protein, was reduced ∆Ig3-MuSK as compared to WT cells. Data are means ± SEM of three biological replicates and independent Western blots (****p < 0.0001, two-way ANOVA with Bonferroni’s multiple comparisons). See also Supplementary Fig. S2 for protein staining of these blots. **F** Cells were treated with 25 ng/ml BMP4 for 2 h, and Car3 transcript levels were assessed. Data are mean ± SEM of 5–6 biological replicates per condition and replicated twice (****p < 0.0001, ***p < 0.001, *p < 0.05, two-way ANOVA with Bonferroni’s multiple comparisons). **G** Cultured primary myotubes from WT and ∆Ig3-MuSK mice were treated with 25 ng/ml BMP4 for 2 h, and levels of Wnt11 mRNA were measured by qPCR. Note that ∆Ig3-MuSK myotubes show reduced Wnt11 expression in response to BMP4 stimulation. Data are means ± SEM from 6 biological replicates from two experiments (****p < 0.0001, two-way ANOVA with Bonferroni’s multiple comparisons)

We first generated stable ΔIg3-MuSK myogenic cell lines to probe MuSK expression and BMP signaling in cells lacking the MuSK Ig3 domain (see “Methods”). These cells also differentiate efficiently into multinucleated myotubes (Supplementary Fig. S1). Immunostaining of unpermeabilized myoblasts with a monoclonal antibody directed against the MuSK Ig2 domain showed that MuSK is expressed at the cell surface and at a comparable level and distribution in both WT and ∆Ig3-MuSK myoblasts (Fig. 2A). MuSK transcript expression is also comparable in WT and ∆Ig3-MuSK cells (Fig. 2B). To probe the role of the MuSK Ig3 domain in the BMP4 response, we serum-starved WT and ∆Ig3-MuSK myoblasts and examined the nuclear localization of pSmad1/5 in the absence or presence of added BMP4. As expected, nuclear pSmad1/5 levels were low at baseline in both genotypes. However, following BMP4 stimulation, the level of nuclear pSmad1/5 was higher in WT compared to ΔIg3-MuSK cells (Fig. 2C). We then performed Western blots to quantify the pSmad1/5 response to BMP4 treatment (Fig. 2D). The ∆Ig3-MuSK cells showed a reduction in BMP4-stimulated pSmad1/5 levels compared to WT (Fig. 2D, E; see also Supplementary Fig. S2). We next assessed the role of the MuSK Ig3 domain in modulating the expression of MuSK-regulated BMP4-induced transcripts Car3 and Wnt11 [15]. Figure 2F shows that levels of BMP4-induced Car3 transcripts are reduced in ∆Ig3-MuSK myoblasts compared to WT. We also confirmed that BMP signaling is perturbed in primary ∆Ig3-MuSK myotubes. We previously showed that cultured MuSK^−/−^ myotubes show reduced BMP-induced Wnt11 expression compared to WT [15]. As shown in Fig. 2G, BMP4-induced Wnt11 transcript levels were reduced in ∆Ig3-MuSK myotubes compared to WT. Finally, to establish that agrin signaling is preserved in ΔIg3-MuSK cells, we assessed agrin-induced AChR clustering in WT and ∆Ig3-MuSK primary myotubes. Agrin-induced AChR clustering was comparable in both genotypes (Supplementary Fig. S3). This normal agrin response in cultured cells is in agreement with the comparable innervation levels observed in WT and ΔIg3-MuSK muscle (Fig. 1G, H). Taken together, these data show that in cultured cells, the MuSK Ig3 domain regulates BMP4 pSmad1/5 and transcriptional responses, and that agrin- and MuSK-BMP-dependent signaling can be clearly distinguished.

### Unique transcriptional profiles in ΔIg3-MuSK fast and slow muscles

**Fig. 3:**
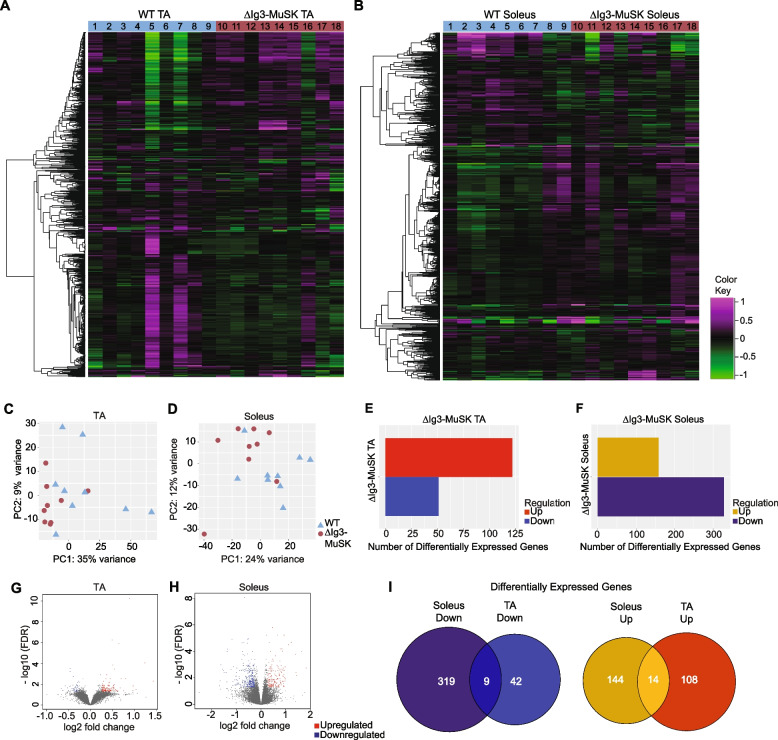
Distinct transcriptomic profiles in ∆Ig3-MuSK TA and soleus. WT and ∆Ig3-MuSK soleus and TA muscles were subjected to RNA sequencing at a depth of ~ 50 million reads per sample (*n* = 9 muscles/genotype; one muscle/animal). Heat map of the top 1000 variable genes in WT and ∆Ig3-MuSK TA (**A**) and soleus (**B**). Each column represents one muscle sample. Principal component analysis of WT and ∆Ig3-MuSK TA (**C**) and soleus (**D**) shows clustering of samples with similar gene expression profiles (▲, WT; ●, ∆Ig3-MuSK; individual muscles). **E** and **F** Differentially expressed genes (DEGs) with 1.2 minimum fold change and adjusted *p*-value < 0.05 were determined using DEseq2 (see also Tables S1, S2). **G** and **H** Volcano plot of -log10 FDR versus gene log2 fold change (red dots up DEGs; blue dots down DEGs). **I** Venn diagram depicting the shared and unique upregulated and downregulated DEGs between ∆Ig3-MuSK TA and soleus

The results from cultured ∆Ig3-MuSK cells indicated that the MuSK Ig3 domain regulates BMP4 signaling and transcriptional response. We next explored the role of the MuSK-BMP pathway in vivo. The soleus muscle expresses several fold higher levels of MuSK transcript compared to the TA [15, 18], suggesting that MuSK may play a particularly important role in this muscle. We compared the transcriptional profiles in 3-month-old WT and ∆Ig3-MuSK TA and soleus using RNA-seq. We isolated RNA from 36 muscles (*n* = 9 TA and soleus muscles per genotype) and sequenced at a depth of ~ 50 million reads/sample. The results revealed striking differences in the transcriptional profiles between these muscles. Figures 3A and B show heatmaps of the transcriptomic profile differences in the 1000 most variable genes in ∆Ig3-MuSK compared to WT in TA and soleus, respectively. Principal component analysis of gene expression profiles showed that both the ∆Ig3-MuSK TA and soleus muscles clustered separately from WT (Fig. 3C, D). Analysis of differentially expressed genes (DEGs) revealed significant changes in both muscles (Tables S1, S2). Genes with adjusted *p*-values < 0.05 showed 122 upregulated and 51 downregulated genes in the TA compared to WT (Fig. 3E), while the soleus exhibited 158 upregulated and 328 downregulated genes (Fig. 3F). The total number of DEGs was 2.8-fold greater in the soleus compared to the TA (Fig. 3G, H). Finally, the identity of DEGs in TA and soleus was strikingly different: only 2.4% (9/370) of the downregulated genes and 5.3% (14/266) of the upregulated genes were shared between TA and soleus (Fig. 3l). Taken together, these results show that the transcriptional profiles of TA and soleus WT and ∆Ig3-MuSK muscles are qualitatively and quantitatively distinct, with the soleus transcriptome being more affected by the downregulation of MuSK-BMP signaling.

**Fig. 4:**
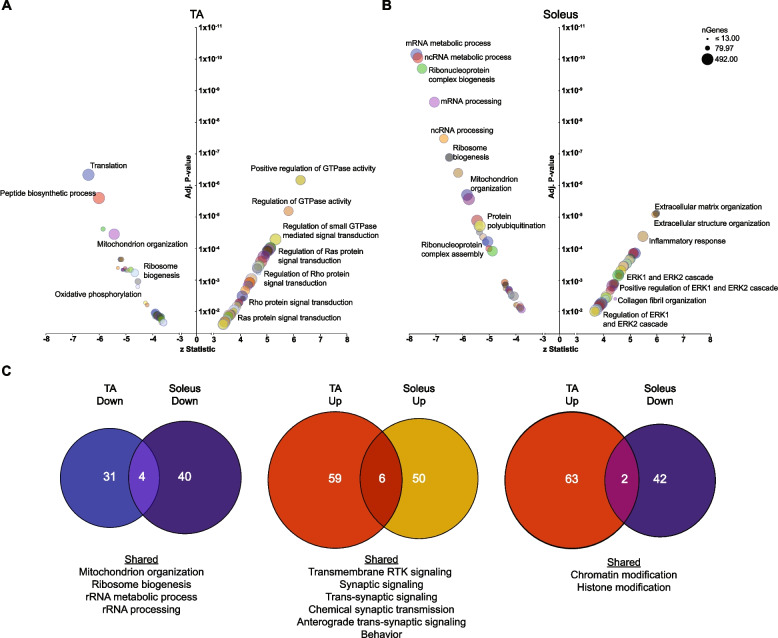
Distinct pathways are dysregulated in ∆Ig3-MuSK TA and soleus. **A** and **B** Gene ontology pathway analysis of top 100 upregulated and downregulated biological process pathways in ∆Ig3-MuSK TA (**A**) and soleus (**B**) (see Table S3). Note the number of highly significant pathways in soleus (adjusted *p*-values between 10^−7^ and 10^−12^) compared to TA. Selective enrichment in soleus down pathways was related to translation including those involving ribosome biogenesis as well as mRNA and ncRNA metabolism. Soleus up pathways included those involved in ECM and inflammation. **C** Venn diagram showing the number of shared and unique downregulated and upregulated pathways between ∆Ig3-MuSK TA and soleus (see Table S3). Note that ≤ 5% of pathways are shared in any of the comparisons. No shared pathways were observed when comparing TA down and soleus up

To gain insight into cellular mechanisms impacted in ∆Ig3-MuSK muscles, we performed gene set enrichment analysis (GSEA) using GO terms for TA and soleus. The specific pathways and the extent of change within pathways differed markedly in TA and SOL (Fig. 4A and B). Multiple upregulated pathways in the TA were related to regulation of GTPase activity and Ras/Rho signal transduction (Fig. 4A, Table S3). In contrast, in the soleus, the major upregulated pathways were related to ECM (extracellular matrix) structure, ECM organization, and inflammation (Fig. 4B, Table S3). In addition, we observed upregulated pathways related to ERK1/2 signaling regulation as well as collagen fibril organization (Fig. 4B). Pathways downregulated in the TA included those related to translation, peptide biosynthesis, protein folding, and ribosome biogenesis (Fig. 4A). Additionally, there were multiple processes related to mitochondrial energy metabolism such as mitochondrial organization, respiratory transport chain, mitochondrial ATP synthesis, and proton transport (Fig. 4A). Most notably, the soleus showed a striking downregulation of biological process pathways related to RNA metabolism including mRNA and ncRNA processing, RNA splicing, and ribonucleoprotein complex biogenesis and subunit organization (all adjusted *p*-values between 10^−6^ and 10^−11^). These changes suggest that the ∆Ig3-MuSK soleus exhibits dysfunctional posttranscriptional RNA metabolism and protein synthesis (Fig. 4B).

Our analysis also revealed a small number (< 5%) of shared GO pathways in TA and soleus, including downregulation of mitochondrion organization and ribosome biogenesis (Fig. 4C). Shared upregulated pathways included synaptic signaling, transsynaptic signaling, chemical synaptic transmission, and anterograde transsynaptic signaling (Fig. 4C), suggesting that although both muscles were fully innervated (Fig. 1F, G), the MuSK-BMP pathway may also play a role at the neuromuscular junction. Some pathways showed opposite directionality, with chromatin organization and histone modification downregulated in soleus while upregulated in the TA (Fig. 4C). Taken together, these analyses suggest that the MuSK-Ig3 domain regulates distinct pathways in ∆Ig3-MuSK TA and soleus.

### Myofiber size is reduced in ∆Ig3-MuSK soleus

**Fig. 5:**
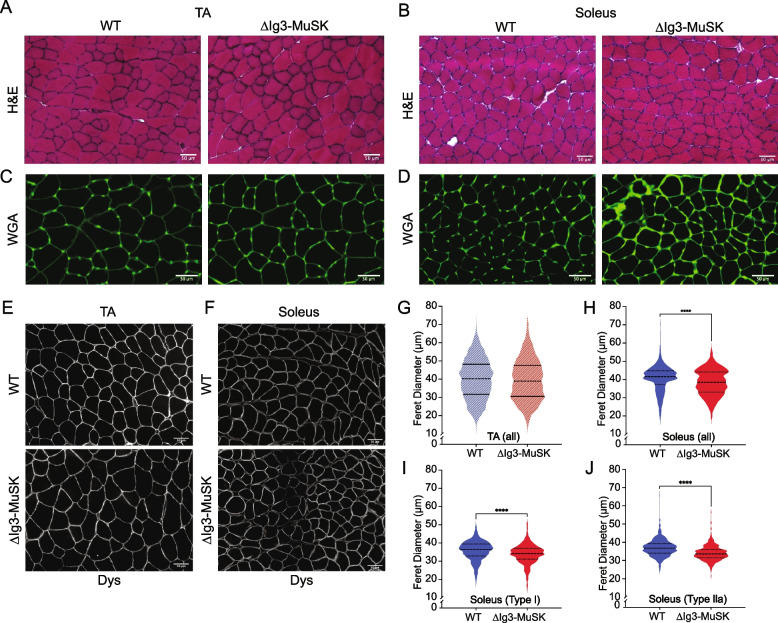
Reduced myofiber size in ∆Ig3-MuSK soleus but not TA. WT and ∆Ig3-MuSK TA and soleus sections were stained with H&E (**A**, **B**), WGA (**C**, **D**), or anti-dystrophin (**E**, **F**). In the TA, the structure (**A**), WGA staining (**C**), and mean Feret diameters (**G**) were indistinguishable in both genotypes. Data are means ± SEM from three animals of each genotype. Average number of muscle fibers counted per animal 963 ± 77. Soleus WT and ΔIg3-MuSK stained with H&E (**B**), WGA (**D**), or dystrophin (**F**). Note that the overall structure of the muscle was comparable, but there is increased interstitial WGA signal in ΔIg3-MuSK soleus. Soleus myofiber Feret diameters of all fibers (**H**), type I fibers (**I**), or type IIa fibers (**J**) were reduced in ΔIg3-MuSK compared to WT. All fibers are as follows: 40.6 µm ± 0.2 WT and 38.4 µm ± 0.2 ∆Ig3-MuSK (697 fibers/animal ± 72, *n* = 3 animals), type I fibers: 36.6 µm ± 0.2 WT and 34.4 µm ± 0.2 ∆Ig3-MuSK (300 ± 33 fibers/animal, *n* = 4 animals), and type IIa: 36.7 µm ± 0.2 WT and 33.9 µm ± 0.2 ∆Ig3-MuSK (487 ± 50, fibers/animal *n* = 4 animals; ****p < 0.0001, Mann–Whitney test)

The muscle-selective reductions in RNA metabolism pathways and ribosome biogenesis raised the possibility that myofiber size is reduced in ΔIg3-MuSK soleus. The overall structure of both the TA and the soleus as revealed by H&E staining was similar in both genotypes (Fig. 5A, B), with no evidence of degeneration observed. The staining levels for dystrophin were comparable in both muscles across genotypes (Fig. 5E, F). Muscle fiber Feret diameter analysis in TA revealed that the myofiber sizes were comparable in ∆Ig3-MuSK and WT (Fig. 5G). In contrast, myofiber sizes were reduced in the ∆Ig3-MuSK soleus (Fig. 5H). This reduction in myofiber size was observed when all myofibers were scored (Fig. 5H) and when either type I (Fig. 5I) or type IIa (Fig. 5J) were separately analyzed using double labeling with myosin-specific markers (see “Methods”). Types I and type IIa myofibers comprise approximately 80% of the myofibers in this muscle [22]. Thus, the large majority of myofiber types in the soleus are reduced in size, while the TA myofiber size is unaffected in ∆Ig3-MuSK mice at this age.

### Increased WGA binding and interstitial collagen in ∆Ig3-MuSK soleus

The GSEA revealed increases in ECM and collagen fibril organization GO pathways (Fig. 4). To confirm and extend this finding, we assessed the ECM histologically. We first used staining with WGA, a lectin that binds a wide range of glycoproteins at the cell surface and is a useful marker for fibrosis [23]. WGA staining in the TA was comparable in both genotypes (Fig. 5C). In contrast, we observed a marked increase in WGA signal in ∆Ig3-MuSK soleus (Fig. 5D). We then examined the distribution and level of type I collagen by immunochemistry in the soleus. Supplemental Figure S4 shows that type I collagen immunostaining is increased in interstitial compartment in ∆Ig3-MuSK compared to WT. Quantification showed that the type I collagen levels were 49% higher in the ∆Ig3-MuSK soleus. These data confirm the GSEA results and suggest that there is fibrosis in the ∆Ig3-MuSK soleus.

### MuSK expression in WT and ∆Ig3-MuSK muscle

**Fig. 6:**
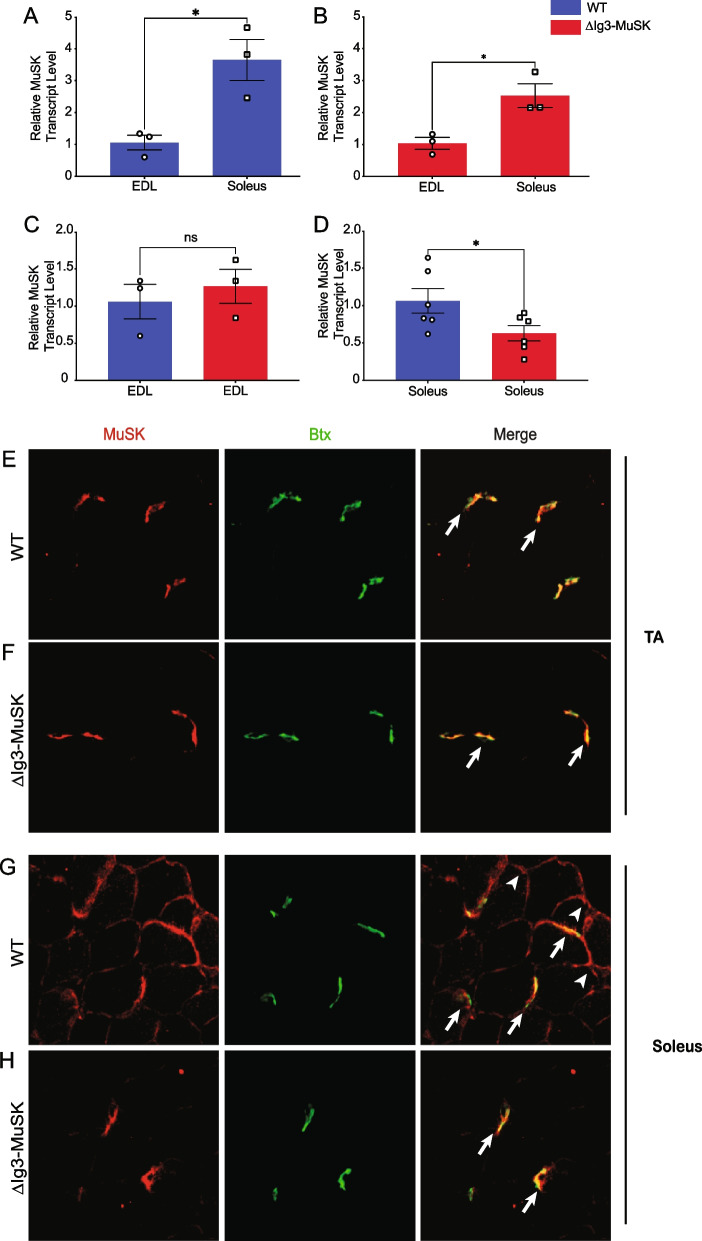
MuSK extrasynaptic localization and transcript expression are reduced in ∆Ig3-MuSK soleus. **A**–**D** MuSK transcript levels in WT and ∆Ig3-MuSK EDL and soleus muscle were quantified by qRT-PCR. Note that MuSK transcript levels are selectively reduced in ∆Ig3-MuSK soleus compared to WT, while the levels in the EDL are comparable in both genotypes. Data are means ± SEM from 3 to 6 different biological replicates (*p < 0.05, unpaired two-tail Student’s *t*-test). **E** and **F** MuSK localization in TA. TA sections from WT and ∆Ig3-MuSK animals were immunolabeled with anti-MuSK (IgG4 serum fraction from MuSK myasthenia gravis patients [24]; 1:1000; red) and α-bungarotoxin (green; to visualize NMJs). In the TA, MuSK is restricted to the NMJ (arrows) in both WT and ∆Ig3-MuSK muscle. No extrajunctional MuSK localization is detected in TA of either genotype. **G** and **H** MuSK localization in soleus. In WT soleus **(G**), MuSK is localized at both the NMJ (arrows) and extrajunctionally (arrowheads). In contrast, in ∆Ig3-MuSK soleus (**H**), MuSK is present at NMJs at levels comparable to WT, but extrajunctional MuSK was not detected

We next examined MuSK mRNA expression and protein localization in WT and ∆Ig3-MuSK muscles. We first assessed the level of MuSK transcripts, which in cultured muscle cells is regulated by the MuSK-BMP pathway [15]. We and others have previously reported that MuSK transcripts are expressed at 3- to 5-fold higher levels in the soleus compared to fast muscles such as EDL and TA [15, 18]. We confirmed these findings in WT EDL and soleus (Fig. 6A). MuSK transcript levels were also higher in soleus as compared to EDL in ∆Ig3-MuSK animals (Fig. 6B). MuSK expression in EDL was comparable in ∆Ig3-MuSK and WT (Fig. 6C). However, MuSK transcript levels in soleus were reduced by ~ 1/2 in ∆Ig3-MuSK compared to WT (Fig. 6D).

We then assessed the localization of MuSK protein. MuSK was localized at all NMJs examined in TA and soleus in both WT and ∆Ig3-MuSK muscle (Fig. 6E–H). In agreement with previous reports [18], in WT muscles, MuSK is localized at extrajunctional domains in the soleus but not in TA [18] (Fig. 6E, G). However, MuSK was not detected in extrajunctional domains in the ∆Ig3-MuSK soleus (Fig. 6H). Taken together, these data indicate that MuSK-BMP signaling is important for regulating the expression and localization of extrajunctional MuSK in the soleus.

### Reduced Akt-mTOR signaling in ∆Ig3-MuSK soleus

We next investigated potential signaling pathways that may mediate the selective size reduction of soleus fibers in ∆Ig3-MuSK mice. The transcriptomic analysis (Fig. 4) showed that multiple RNA metabolism pathways were selectively downregulated in the ∆Ig3-MuSK soleus. These observations suggested that decreased protein synthesis could underlie the reduced myofiber size observed in the soleus. One candidate pathway is IGF1-Akt-mTOR, which plays a central role in regulating muscle growth [25, 26]. Moreover, several members of this pathway are significantly dysregulated in the ∆Ig3-MuSK soleus but not the TA including Igf1, Igf2bp2, Igfbp2, and Txnip (Supplemental Table S4). Other members of this pathway, including IRS1, IRS2, rheb, and the antisense lncRNA Gm15441 to TXNIP, also showed strong trends in dysregulation in the ∆Ig3-MuSK soleus (*p* = 0.051–0.065), but not in the TA.

**Fig. 7:**
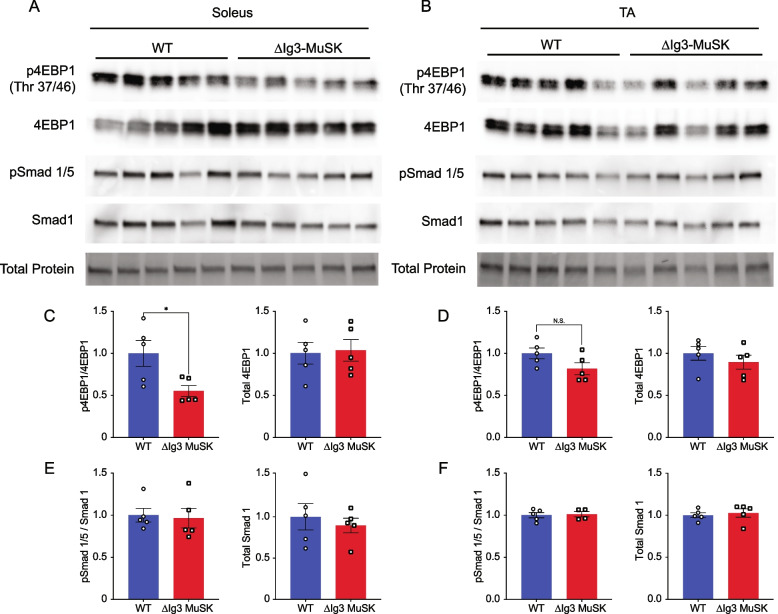
Akt-mTOR signaling is reduced in ∆Ig3-MuSK soleus but not TA. **A** WT and ∆Ig3-MuSK soleus muscle protein extracts were isolated for Western blotting of pSmad1/5, total Smad1, p-4EBP1, and total 4EBP1. Total protein stain was used as loading control and for protein normalization. Levels of pSmad1/5 and p4EBP1 were determined as a ratio to total Smad1 and total 4EBP1, respectively. Note that p-4EBP1 levels were reduced in ∆Ig3-MuSK soleus compared to WT (**C**) but unchanged in TA (**D**). pSmad 1/5 levels in ∆Ig3-MuSK soleus and TA were comparable to WT (**E** and **F**). Data are means ± SEM from five biological replicates (*p < 0.05, unpaired two tailed Student’s *t*-test)

To directly assess the activity of the Akt-mTOR pathway in ∆Ig3-MuSK mice, we measured the phosphorylation of members this pathway in TA and soleus. Translation is promoted by phosphorylated 4EBP1 (p4EBP1) and is inhibited by the unphosphorylated form (4EBP1). In a first set of experiments (see “Methods”), we probed Western blots of muscle extracts from soleus and TA muscles of each genotype with antibodies against 4EBP1 and p4EBP1 (Fig. 7A, B). Quantification showed that p4EBP1 levels were reduced by > 40% in the ∆Ig3-MuSK soleus (1 ± 0.15 and 0.55 ± 0.06, ± SEM in WT and ∆Ig3-MuSK, respectively; *p* = 0.02; *n* = 5 muscles/genotype) (Fig. 7C; Supplementary Fig. S5). In contrast, p4EBP1 levels were comparable in WT and ∆Ig3-MuSK TA (Fig. 7D). Notably, pSmad 1/5 levels did not differ in ∆Ig3-MuSK compared to WT for both the TA and soleus (Fig. 7E, F; see “Discussion”). To confirm and extend these observations, we performed an independent set of experiments in a second laboratory using muscles from a different cohort of mice (see “Methods”). In agreement with the first set of experiments (Fig. 7), p4EBP1 levels were reduced in the soleus (Supplementary Fig. S6). Importantly, in this experiment, S6 phosphorylation status was also probed. As shown in Fig. S6, the levels of P-S6 were also reduced in the soleus. pAkt levels were unaffected. P-S6 and p4EBP1 are the two main effectors of the Akt-mTOR pathway that regulate protein synthesis. Thus, taken together with the transcriptomic analysis, these results establish that Akt-mTOR pathway activity is selectively reduced in ∆Ig3-MuSK soleus and provide a mechanistic basis for the decreased myofiber size observed in this slow muscle.

## Discussion

In this report, we introduce the MuSK-BMP pathway as a novel regulator of myofiber size in slow muscle. This pathway is selective for the slow soleus muscle as compared to the predominantly fast TA. In ∆Ig3-MuSK mice, the soleus is reduced in size, RNA metabolic pathways are downregulated, and Akt-mTOR signaling is reduced compared to TA. Our findings indicate that the locus of MuSK-BMP action is extrasynaptic throughout the soleus, rather than secondary to changes at the NMJ. These results also reveal a novel in vivo function for MuSK that is independent of its role in agrin-LRP4 signaling at the synapse. The findings shed light on the mechanism for the muscle-selective regulation of myofiber size and reveal a new pathway for promoting muscle growth and combatting atrophy.

Our results demonstrate that deletion of the MuSK Ig3 domain selectively perturbs MuSK function as a BMP co-receptor. First, MuSK protein lacking the Ig3 domain is localized at the cell surface in cultured ∆Ig3-MuSK myogenic cells, and the levels of MuSK mRNA are comparable in cultured ∆Ig3-MuSK and WT cells (Fig. 2). ∆Ig3-MuSK is also localized at all NMJs examined in vivo (Fig. 6; see below). This high-fidelity expression and localization are consistent with the fact that the ∆Ig3-MuSK allele created by gene editing mimics a natural MuSK splice isoform [21, 27]. Second, cultured ∆Ig3-MuSK myogenic cells show reduced levels of pSmad 1/5 signaling and target gene expression in response to BMP treatment (Fig. 2). Notably, the MuSK-regulated BMP-induced transcripts include Wnt11 and Car3, which were previously identified in a study using cultured MuSK^−/−^ myogenic cells [15]. In contrast, the agrin-LRP4-mediated functions of MuSK, which require its Ig1 domain, are spared: agrin-induced AChR clustering is robust in cultured ∆Ig3-MuSK myotubes (Supplementary Fig. S3); in vivo, NMJ innervation levels and grip strength are comparable to WT in these 3-month-old mice (Fig. 1).

Our transcriptomic, morphological, and biochemical results show that the MuSK-BMP pathway plays a selective role in slow as compared to fast muscle. The MuSK-BMP pathway regulates myofiber size in slow muscle. In the ∆Ig3-MuSK soleus, reduced size was observed in both types I and IIa fibers (Fig. 5), which together comprise ~ 80% of fibers in this muscle. In contrast, no significant differences were observed in the diameter of TA myofibers, which are predominantly type IIb, in the 3-month age animals examined here (Fig. 5). The sets of both the up- and down-regulated genes in soleus and TA were also remarkably distinct. Only 23/663 DEGs were shared between soleus and TA (Fig. 3). GO pathway analysis also revealed distinct functions for the MuSK-BMP pathway in soleus and TA (Fig. 4; Table S3). As discussed below, a large number of downregulated GO pathways involved in RNA metabolism were unique to soleus. However, it is noteworthy that the two shared downregulated GO pathways were related to mitochondria organization and ribosome biogenesis, which raises the possibility that the MuSK-BMP pathway may regulate energy metabolism and some aspects of protein synthesis in both fast and slow muscle. These shared pathways could also reflect the contribution of the small number (~ 15%) of IIa fibers in the TA. We note that the reduced myofiber size in soleus observed at 3 months of age could be due to developmental processes or alternatively reflect an atrophy of mature fibers.

**Fig. 8:**
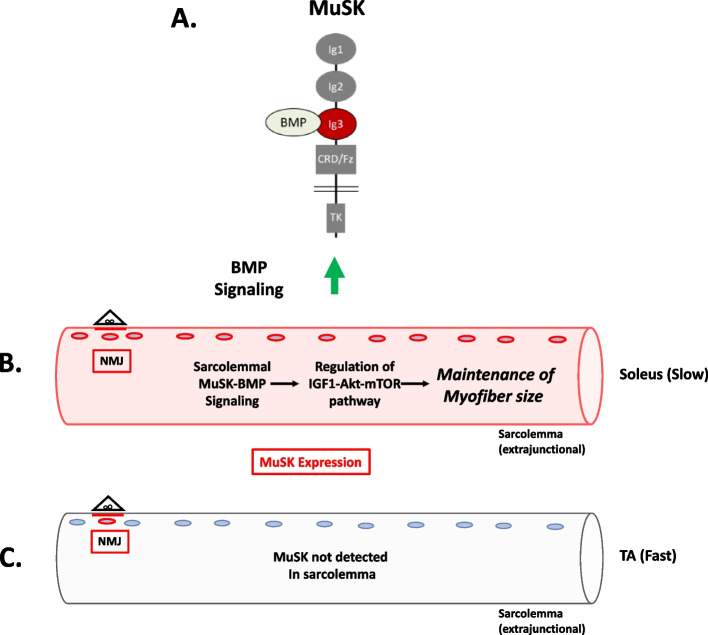
A model for MuSK-BMP regulation of muscle fiber size in slow muscle. **A** MuSK containing the Ig3 domain binds BMP and promotes BMP signaling. **B** In soleus, MuSK is expressed at the sarcolemma and regulates myofiber size via the Akt-mTOR pathway. **C** MuSK is not detected in the sarcolemma in fast muscles (e.g., TA, EDL, STM). Note that MuSK is expressed at the NMJ of all muscles. We propose that MuSK-BMP signaling in the sarcolemma selectively regulates myofiber size in slow muscle by controlling the IGF1-Akt-mTOR pathway

Several lines of evidence indicate that the MuSK-BMP pathway maintains soleus myofiber size through the regulation of the IGF1-Akt-mTOR pathway, the primary anabolic regulator of muscle cell size (Fig. 8) [25, 28–33]. This pathway increases protein synthesis through mTOR-mediated phosphorylation of key elements regulating translation, notably 4EBP1 and S6. Our transcriptomic analysis revealed a host of downregulated GO pathways in RNA metabolism as well as dysregulation of members of the IGF1-Akt-mTOR pathway that were selective for the soleus. Importantly, biochemical analysis showed that both p4EBP1 and P-S6, direct targets of mTOR, are downregulated in ∆Ig3-MuSK soleus (Fig. 7; Supplementary Fig. S6). Notably, others have observed muscle-selective effects of the mTOR inhibitor rapamycin on regulating myofiber size, where it has also been linked to reductions in RNA metabolism components [32, 34]. We saw no evidence that this reduced myofiber size was due to denervation, since the NMJs in both muscles were fully innervated and grip strength is comparable to WT (Fig. 1). Further, our transcriptomic analysis detected few signatures of upregulated protein degradation, such as the atrogenes (Table S5), which are markedly upregulated following denervation, immobilization, or cachexia [35, 36]. Taken together, our results support a model where the MuSK-BMP pathway maintains muscle mass by regulating protein translation through modulation of the Akt-mTOR pathway (Fig. 8).

The striking selectivity of the MuSK-BMP pathway in the soleus as compared to the TA is likely a function of the distinct expression, localization, and regulation of MuSK in this muscle. MuSK transcript levels are ~ 3– 5-fold higher in the WT soleus compared to the fast EDL (Fig. 6). MuSK is present at NMJs in all muscles [32, 37], including TA and soleus (Fig. 6). However, in WT soleus, MuSK is also localized at extrasynaptic domains along the extent of the myofiber (Fig. 6), which is in agreement with earlier reports [15, 18]. Further, snRNAseq analysis shows robust MuSK expression in soleus as compared to TA myonuclei [38]. Importantly, both MuSK transcript levels and the localization of MuSK at extrasynaptic domains are selectively reduced in ∆Ig3-MuSK soleus. This reduction seems likely to be the result in part of perturbed autoregulation since MuSK itself is a MuSK-BMP-dependent transcript [15]. On the organismal level, our results suggest that MuSK expression in the sarcolemma may be one mechanism conferring muscle-selective regulation of myofiber size in health and disease.

Our results add a novel dimension to our understanding of the role of BMP signaling in regulating muscle size. Previous studies have shown that increasing BMP signaling by overexpression of BMP7 or constitutively active BMPR1a (ALK 3) causes hypertrophy. Notably, the hypertrophy is blocked by the mTOR inhibitor rapamycin, establishing a link between BMP signaling and Akt-mTOR-mediated muscle growth [10]. These results also align with our observation that this pathway is an important output of MuSK-BMP signaling (Fig. 7, Supp. Fig. S6). It is of interest that while BMP-stimulated pSMAD signaling is reduced in cultured ∆Ig3-MuSK (Fig. 2) or MuSK^−^/^−^ [15] myogenic cells, we did not observe decreased pSMAD signaling in extracts of either soleus or TA (Fig. 7). We also did not observe changes in the BMP signaling pathway members in our transcriptomic analysis (Supp. Tables S2 and S3). This finding could reflect low MuSK-BMP pSMAD activity in the steady-state condition present in mature muscle and/or a masking of muscle pSMAD signal by MuSK-independent pSMAD signaling in non-myogenic cells, which comprise > 50% of nuclei in muscle [38, 39]. It is also possible that noncanonical BMP signaling pathways [40–42] mediate some of the effects observed here. Elucidating their potential role will be the focus of future experiments.

Our findings also point to other functions of the MuSK-BMP pathway in muscle. The increase in WGA binding and type I collagen expression in the ∆Ig3-MuSK soleus indicates that there is a low level of fibrosis in this muscle. Fibrosis is often the consequence of muscle damage. Thus, the MuSK-BMP pathway may play a role in maintaining the integrity of slow muscle. The soleus-selective increase in inflammatory pathways observed in the GO pathway analysis is also consistent with this interpretation. However, we observed no evidence of myofiber degeneration/regeneration (e.g., centrally nucleated fibers) in either soleus of TA, indicating that the level of damage to the myofiber in the ∆Ig3-MuSK is subtle. Finally, we also observed a number of elevated GO pathways related to synaptic signaling and organization in both TA and soleus. This observation raises the possibility that the MuSK-BMP pathway, while not essential for synapse formation and maintenance, also plays a role at the NMJ. Studies are in progress to address this question.

The role of MuSK in maintaining muscle size has potential implications for myasthenia gravis (MG) caused by autoantibodies to MuSK (“MuSK-MG”) [43]. This form of MG is distinct from the more common anti-AChR MG, can cause different symptoms, and does not respond to cholinesterase inhibitors. The pathogenesis of MuSK-MG is mediated at least in part by IgG4 antibodies directed against the MuSK Ig1 domain that disrupt agrin-LRP4 binding and signaling [17, 24, 44–46]. However, some clinical features of MuSK-MG suggest that non-synaptic pathology mediated by the MuSK autoantibodies may also contribute to the disease. MuSK-MG pathology is often more pronounced in restricted muscle groups, including bulbar and respiratory muscles [47, 48]. Moreover, muscle atrophy is observed in MuSK-MG where it is associated with non-fluctuating weakness, fatty tissue infiltration, and myopathic changes in electrophysiological activity. It is therefore plausible that antibodies targeting the MuSK expressed in the sarcolemma could contribute to MuSK MG pathology.

The MuSK-BMP pathway could also be a target for promoting muscle growth and treating conditions associated with muscle atrophy such as sarcopenia, immobilization, and cachexia. Maintenance of muscle mass is a balance between the homeostatic mechanisms regulating protein synthesis and degradation. Although the role for IGF1 as an anabolic pathway is well established, circulating IGF1 levels correlate incompletely with muscle status. Rather, muscle-derived IGF1 is likely to be the dominant mediator of growth [33]. The MuSK-BMP pathway represents an attractive target for developing specific agents to modulate muscle growth. This pathway also offers prospects for the precise manipulation of BMP signaling in muscle. BMPs and their canonical receptors are ubiquitous, and manipulating them leads to unwanted side effects; in contrast, MuSK expression is highly enriched in muscle. Moreover, the MuSK ectodomain would be accessible to manipulation by therapeutic antibodies, while antisense oligonucleotides could promote MuSK-BMP signaling without affecting the role of MuSK in synapse formation. Finally, MuSK levels increase with age in humans and rats [38, 49, 50]. The MuSK-BMP pathway thus emerges as an attractive target for selectively modulating muscle growth and combatting atrophy.

## Materials and methods

### Animals

To target the MuSK Ig3 domain, we deleted exons 6 and 7 using CRISPR-Cas9 (exon numbering according to ENSMUST00000081919; Fig. 1) in C57BL/6 mice sourced from The Jackson Laboratory (JAX). Founder mice carrying the MuSK^∆Ig3^ allele were backcrossed to wild-type (WT) C57BL/6 background mice, and confirmation of germline transmission of the MuSK^∆Ig3^ to progeny was performed by PCR using isolated genomic DNA. PCR for WT and ∆Ig3-MuSK was performed using DreamTaq Green PCR Master Mix (Thermo Scientific) according to manufacturer’s directions using 60 °C annealing temperature and 60-s extension for 33 cycles. WT MuSK primers used were forward: TGGGCACTCAATCCAGCAG and reverse: TGGCTAAGCAAGGCAGGAC. ∆Ig3-MuSK primers used were forward: TGGGCACTCAATCCAGCAG and reverse: TGGTATCCATCACTTGAACAAG. Offspring were backcrossed to WT C57BL/6 for several generations. Mice of 3 months of age were used for all experiments. All protocols were conducted under accordance and approval of the Brown University Institutional Animal Care and Use Committee.

### Generation of immortalized ΔIg3-MuSK myogenic cell lines

WT and ∆Ig3-MuSK mice were crossed with the H-2 Kb-tsA58 transgenic Immortomouse mouse line (JAX cat. no. 032619), and immortalized myoblast cell lines were established as described previously [51, 52]. Myoblasts were isolated from postnatal day 2–3-old pups, and cells were subcloned on Matrigel for 1–2 passages and then moved to gelatin-coated plates for all future passages [53]. Only clones that were WT and ∆Ig3-MuSK homozygous and containing the H-2 Kb-tsA58 transgene were maintained. Cells were cultured at 33 °C in 8% CO_2_ in Dulbecco’s modified Eagle’s medium supplemented with 20% fetal bovine serum, 2% L-glutamine, 1% penicillin–streptomycin, 1% chicken embryo extract, and 1 U of γ-interferon. For primary cultures, myoblasts were isolated as described previously [53] and maintained in growth media consisting of Iscove’s modified Dulbecco’s medium (IMDM) containing 20% FBS, 1% CEE, and 1% penicillin–streptomycin and differentiated in IMDM supplemented with 2% horse serum and 1% penicillin–streptomycin when myoblasts reached 70% confluency. For BMP4 treatments, cells were serum starved 5 to 6 h (myoblasts) or were maintained in low-serum differentiation conditions for 24–48 h (myotubes) and then treated with 20–25 ng/ml recombinant human BMP4 protein (R&D Systems, cat. no. 314-BP). For acetylcholine receptor clustering experiments, primary myoblasts were isolated and differentiated into myotubes as in [53]. Myotubes were stimulated with 10 units of agrin (R&D Systems, cat. no. 550-AG) for 16 h and stained with rhodamine-conjugated α-bungarotoxin (Thermo Fisher, cat. no. T1175) at 1:2000. AChR clusters were counted as previously described [54].

### Histology, immunohistochemistry, and immunocytochemistry

Muscles were flash frozen in freezing isopentane, embedded in optimum cutting temperature media, and cryosectioned at 10 µm. For histological analysis, sections were stained with hematoxylin and eosin. To delineate muscle cell membrane and ECM, sections were stained with wheat germ agglutinin (WGA; Themo Fisher cat. no. 11261) conjugated to Alexa Fluor 488 in accordance with Treat-NMD standard operating procedures (SOP ID MDC1A_M.1.2.002). For muscle fiber sizing experiments, sections of WT and ∆Ig3-MuSK TA and soleus muscles were rehydrated and blocked in 20% goat serum then incubated with anti-dystrophin diluted to 1:400 overnight and detected with goat anti-rabbit IgG Alexa Fluor 488 (Invitrogen, cat. no. 11034) secondary antibodies. To identify types I and IIa muscle fibers, a cocktail of anti-dystrophin and mouse IgG1 monoclonal SC-71 (type IIa) or IgG2b BA-D5 (type I) was used at 1:100 and detected with fluorescent serotype-specific secondaries. Fiber sizes were measured using the open-source QuantiMus plug-in [55]. Muscle sections were stained for MuSK using an IgG4 serum fraction (1:1000) from MuSK myasthenia gravis patients containing MuSK autoantibodies targeting the MuSK Ig1 domain [56] (a gift from M. Huijbers, Leiden University). Sections were fixed with 1% PFA, washed, and blocked with 2% BSA and 5% goat serum in PBS. Serum was diluted 1:1000 in 1/10 block overnight at 4 °C and detected using goat anti-human IgG Alexa Fluor 555 (Invitrogen) diluted 1:1000 and counterstained with Alexa Fluor488-conjugated α-bungarotoxin (Thermo Fisher, cat. no. 11013) at 1:1000. WT and ∆Ig3-MuSK myoblasts were stained for phospho-Smad1/5 (Ser463/465; CST no. 9516) according to the manufacturer’s protocol. For MuSK staining in cells, unpermeabilized myoblasts were fixed in 1% PFA and immunolabeled with human anti-MuSK monoclonal antibodies targeting the MuSK Ig2 domain (a gift from K. O’Connor, Yale University [57], and detected with goat anti-human IgG AlexaFlour 555 (Thermo Fisher, cat. no. A-21433). For myosin heavy chain staining of myotubes, cultures were fixed and permeabilized with ice-cold methanol, blocked with 2% BSA, incubated with anti-pan myosin heavy chain A4.1025 (Developmental Studies Hybridoma Bank; contributed by H.M. Blau), and followed by Alexa Fluor488-conjugated anti-mouse secondary antibody.

Images were acquired on a Nikon Ti2-E inverted microscope equipped with a Photometrics Prime 95B sCMOS camera for fluorescence imaging and a 16-megapixel Nikon DS-Ri2 color camera for imaging histology slides. Confocal z-stack images were obtained using a Zeiss 800 LSM laser scanning microscope equipped with a USRB laser module and GaAsP detectors. When comparing fluorescence levels, all images were acquired on the same session and imaging parameters. Images were processed using ImageJ (NIH).

For collagen, immunostaining sections were incubated with the primary antibody rabbit anti-Collagen 1 alpha 1 as described above. For quantification of fluorescence intensity, we used ImageJ to manually trace the periphery of myofiber and measured the mean intensity of Collagen1 α1. Background intensity was acquired from the center of the same myofiber bearing the edge. The mean intensities of Collagen 1 alpha 1 minus the background of C57BL/6 and ΔIg3-MuSK soleus muscles were compared with an unpaired *t*-test in Prism. Three segments of different myofibers were analyzed for five sections of C57BL/6 and ΔIg3-MuSK soleus muscles (*n* = 6).

For visualization of NMJs in whole mount preparations, muscles were collected and pinned at resting length for removal of connective tissue, fixed in 4% PFA for 20 min, fileted into bundles, washed with PBS 3 × 10 min, and labeled with tetramethylrhodamine-conjugated ⍺-bungarotoxin (1:40, Invitrogen T1175) for 15 min at room temperature. After washing, muscles were incubated in methanol at − 20 °C for 5 min. Following washing tissue was blocked for 1 h in 0.2% Triton X-100 with 2.0% bovine BSA in PBS and then incubated with primary antibodies (rabbit anti-neurofilament; 1:2000, Sigma-Aldrich AB1987) and rabbit anti-VAChT (1:500, Synaptic Systems, cat. no. 139103) overnight with gentle agitation at 4 °C. Muscles were washed 3 × 10 min and incubated in Alexa Fluor goat anti-rabbit 488 (1:200, Invitrogen A11008) for 4 h and mounted in VECTASHIELD Mounting Medium with DAPI (Vector H-1200–10). Slides were blinded before imaging. Images were obtained using a Zeiss LSM 800 confocal microscope using a 40 × objective with optical sections taken at 2-µm intervals, using Zeiss Zen Blue software. NMJ morphometry was assessed using the aNMJmorph macro [58]. Innervation was assessed by comparing the percentage of AChR overlapped by presynaptic staining, normalized to WT overlap.

### Western blotting

Western blotting was performed independently in two laboratories. For site 1 (Figs. 2 and 7), samples were homogenized in ice-chilled RIPA buffer (ThermoFisher), cOmplete® EDTA-free protease and phosphatase inhibitor cocktail (Roche) supplemented with 1-mM sodium orthovanadate, 10-mM sodium fluoride, and 1-mM EDTA. Homogenates were centrifuged at 10,000 × g for 10 min at 4 °C. Total protein concentration of clarified supernatants was determined using the Bradford reagent (ThermoFisher) and BSA standard (ThermoFisher). Equal amounts of protein were separated by SDS-PAGE using 4–15% gels (Bio-Rad) and transferred to nitrocellulose membranes using a tank transfer system. Total protein loading for transfer efficiency and normalization was assessed using no-stain protein labeling reagent (Invitrogen). Membranes were blocked in 5% nonfat dry milk and probed with primary antibodies overnight according to manufacturer’s recommended protocols. All primary antibodies used were rabbit monoclonals obtained from Cell Signaling Tech: phospho-Smad1/5 (Ser463/465) (#9516), total Smad1 (D5957, #6944), phospho-4E-BP1 (Thr37/46) (236B4; #2855), and total 4E-BP1 (53H11, #9644). Primary antibodies were detected with goat anti-rabbit Fc fragment-specific HRP-conjugated IgG (Jackson ImmunoResearch, cat. no. 111–035-046) used at 1:5000. Membranes were visualized by chemiluminescence and under Epi-Blue to visualize total protein no-stain labeling using a ChemiDoc MP imaging system (Bio-Rad). Densitometric analysis was performed using ImageLab software (Bio-Rad).

For site no. 2 (Supp. Fig. S6), the method was as described previously [59]. Briefly muscle was homogenized in 80 mL of buffer containing 50-mM Tris (pH 7.5), 150-mM NaCl, 10-mM MgCl2, 0.5-mM DTT, 1-mM EDTA, 10% glycerol, 2% SDS, 1% Triton X-100, Roche Complete Protease Inhibitor Cocktail, and Roche Phospho-Stop Phosphatase Inhibitor Cocktail. Lysates were incubated at 70 °C for 10 min and then centrifuged at 13,500 rpm for 15 min at 4 °C, and protein concentration was measured using BCA Protein Assay Kit (Pierce). Fifty milligrams of the lysate was run on a precast 4–12% SDS polyacrylamide gel (Invitrogen, NW04122BOX). After transfer, nitrocellulose membrane blocking was performed in 5% nonfat dry milk for 1 h at room temperature followed by the primary antibody incubation over night at 4 °C. Secondary antibody incubation was performed for 1 h at room temperature. Detection was performed using Immobilon Classic Western HRP Substrate (Millipore, WBLUC0500) using the UVITEC ATOM imaging system. Antibodies are as follows: Cell Signalling pAKT (S473; Ref. 4060), P-S6 (S240/244; Ref. 5364), S6 (Ref. 2217), AKT (Ref. 9272), p4E-BP1 and 4E-BP1 as above, and Abcam GAPDH (Ref. 8245). Images were quantified using ImageJ software, and data were normalized by Ponceau total protein.

### Quantitative RT-PCR

Total RNA from snap frozen muscles and cells stored in RNAlater (Invitrogen) was isolated using the RNeasy Fibrous and RNeasy Mini Kit (Qiagen), respectively, and DNase I treated according to manufacturer’s protocol. cDNA from total RNA was reverse transcribed using SuperScript III cDNA Synthesis Kit (Invitrogen) and analyzed by quantitative RT-PCR using TaqMan assays (Applied Biosystems) targeting MuSK (Mm00437762_m1), Wnt11 (Mm00437327_g1), Car3 (Mm00775963_g1), and Id1 (Mm001281795_m1). Data was analyzed by ∆∆Ct method using villin-like (Mm00457074_m1) and 18S (Hs99999901_s1) reference genes for muscle and cultured cells, respectively, which were expressed in at comparable levels in the WT and ∆Ig3-MuSK samples (see Supp. Fig. S7).

### RNA-seq analysis

Total RNA from WT and ∆Ig3-MuSK mouse soleus and TA was extracted using a RNeasy Fibrous Tissue Mini Kit (Qiagen) for non-stranded RNA library preparation and sequencing (Genewiz). Samples were sequenced at a depth of approximately 50 million reads/sample. Reads were assessed for quality and trimmed of adapter sequences using Trimmomatic and then aligned with GSNAP to the Ensembl mouse reference genome (mm10), and read count matrices were generated using htseq-count. WT and ∆Ig3-MuSK read count matrices were uploaded to the integrated Differential Expression and Pathway (iDEP) analysis tool for exploratory data analysis, differential gene expression using DEseq2, and gene set enrichment pathway GO analysis [60]. Genes with a 1.2 minimum fold change and 0.05 false discovery rate (FDR) were considered as differentially expressed. Gene set enrichment analysis was performed in a cutoff-free manner with GSEA to screen for “enriched” MSigDB GO and canonical pathway annotation gene sets [61]. Pathways with an adjusted *p*-value of < 0.05 were considered for further analysis. The raw datasets are deposited in GEO GSE212352.

### Statistical analysis

The average of biological replicates is shown as mean ± SEM. Experiments were replicated two to three times as indicated. Statistical comparisons between groups were performed using two-way ANOVA and unpaired Student’s *t*-test when comparing multiple groups or two groups respectively. ANOVA analyses were corrected by post hoc test as indicated. Significance was determined as *p* < 0.05 (***p < 0.001, **p < 0.01, *p < 0.05).

### Supplementary Information

**Additional file 1: Supplementary Fig. S1.** Immortalized WT or ∆Ig3-MuSK  myoblasts were cultured in differentiating conditions for 3 days. The cultures were labeled with the pan-skeletal muscle myosin anti-MyHC (green; see “Methods”) and DAPI as described in methods. Note that ∆Ig3-MuSK cells differentiated into multi-nucleated myotubes that expressed MyHC. **Supplementary Fig. S2.** Supporting data for Western Blot in Fig. 2D showing total protein visualized using the No-Stain reagent (see “Methods”). **Supplementary Fig. S3.** Agrin-induced AChR clustering is comparable in WT and ∆Ig3-MuSK myotubes. WT and ∆Ig3-MuSK cultured primary myotubes were treated with agrin for 16 hr. (A) Visualization of AChR distribution. AChR clusters are denoted by arrows. (B) Quantification of AChR clusters. The agrin response was comparable in WT and ∆Ig3-MuSK  myotubes (two-way ANOVA with Bonferroni’s multiple comparisons). **Supplementary Fig. S4.** Increased type I collagen levels in ∆Ig3-MuSK soleus. Sections of 3-month-old soleus muscle from WT and ∆Ig3-MuSK  were stained with antibodies to Type I Collagen (red) and DAPI (blue). (A) Imaging. Note the increase in interstitial collagen levels in the mutant muscle compared to WT. (B) Quantification showed that Type I collagen levels were increased by 49% in the ∆Ig3-MuSK soleus (577.6 ± 33.7, *n*=47 and 860.6 ± 37.3, *n*=45 in WT and ∆Ig3-MuSK respectively, ****p< 0.0001, unpaired t-test; *n*=6 muscles per genotype; 5-6 sections/muscle. **Supplementary Fig. S5.** Supporting data for Western Blot in Fig. 7A (Soleus) and 7B (TA) showing total protein staining (Ponceau).  **Supplementary Fig. S6.** P-S6 and p4EBP1 are down-regulated in ∆Ig3-MuSK soleus. Homogenates of  3-month-old soleus muscle from a different cohort of mice than used in Fig. 7 (see “Methods”) were separated by SDS-PAGE and probed with the indicated antibodies to phosphorylated (‘p’; A) or unphosphorylated (B) 4EBP1, S6, or Akt.  Both blots were probed for GAPDH as a loading control. Total protein (Ponceau stain; C,D) of the same blots obtained prior to the antibody incubations (*N*= 5 WT and 5 ∆Ig3-MuSK mice, * *p*<0.05). Note that the levels of both p4EBP1 and P-S6 were downregulated in the ∆Ig3-MuSK soleus.  **Supplementary Fig. S7.** Expression levels of the reference genes used for qRT-PCR for myoblasts (A; 18S ribosomal RNA); and for soleus (B) and EDL (C) muscles (Villin-like mRNA). Note the comparable expression between genotypes. 

**Additional file 2: Table S1.** Differentially-expressed genes in WT and ∆Ig3-MuSK TA. **Table S2.** Differentially-expressed genes in WT and ∆Ig3-MuSK Soleus. **Table S3.** GO terms for ∆Ig3-MuSK soleus and TA. **Table S4.** Dysregulated Akt-mTOR pathway genes in the soleus compared to TA. **Table S5.** Atrogenes expression in WT and ∆Ig3-MuSK soleus and TA muscle.

## Data Availability

The raw RNA-seq datasets are available in GEO GSE212352. Access to other primary data and experimental resources will be available on request.
