# Supplementary figures and images for: The MuSK-BMP pathway maintains myofiber size in slow muscle through regulation of Akt-mTOR signaling
Source: Skelet Muscle. 2024 Jan 3;14:1. doi: 10.1186/s13395-023-00329-9 (PMC10763067; doi:10.1186/s13395-023-00329-9)

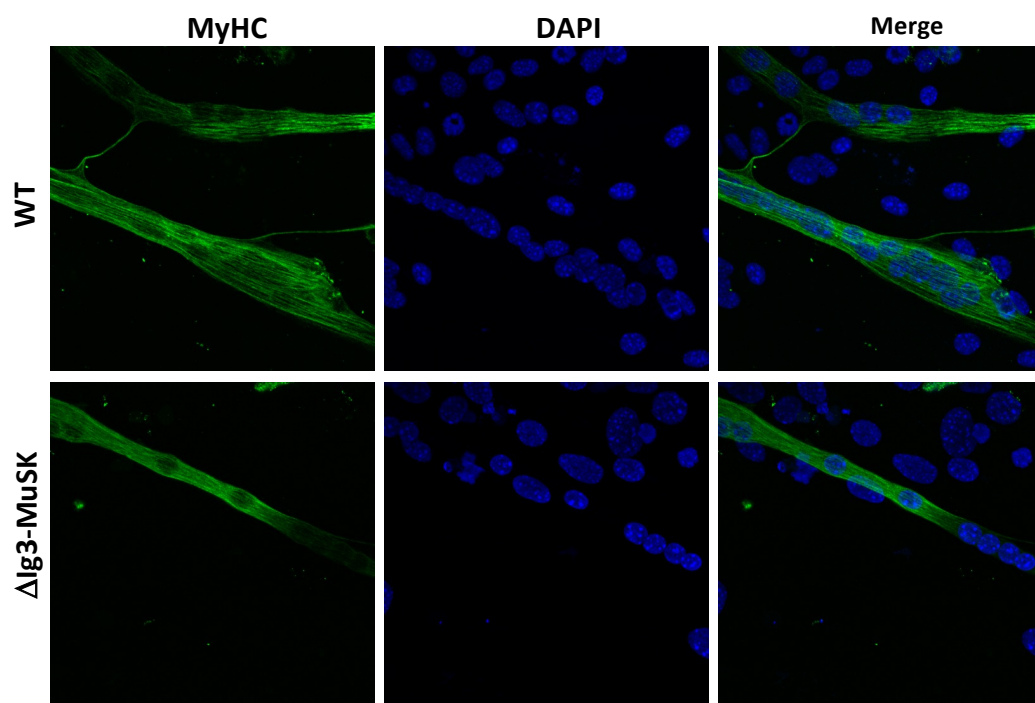

Supplementary Figure S1.

Supplement: Supplementary file 1 — Additional file 1: Supplementary Fig. S1. Immortalized WT or ∆Ig3-MuSK myoblasts were cultured in differentiating conditions for 3 days. The cultures were labeled with the pan-skeletal muscle myosin anti-MyHC (green; see “Methods”) and DAPI as described in methods. Note that ∆Ig3-MuSK cells differentiated into multi-nucleated myotubes that expressed MyHC. Supplementary Fig. S2. Supporting data for Western Blot in Fig. 2D showing total protein visualized using the No-Stain reagent (see “Methods”). Supplementary Fig. S3. Agrin-induced AChR clustering is comparable in WT and ∆Ig3-MuSK myotubes. WT and ∆Ig3-MuSK cultured primary myotubes were treated with agrin for 16 hr. (A) Visualization of AChR distribution. AChR clusters are denoted by arrows. (B) Quantification of AChR clusters. The agrin response was comparable in WT and ∆Ig3-MuSK myotubes (two-way ANOVA with Bonferroni’s multiple comparisons). Supplementary Fig. S4. Increased type I collagen levels in ∆Ig3-MuSK soleus. Sections of 3-month-old soleus muscle from WT and ∆Ig3-MuSK were stained with antibodies to Type I Collagen (red) and DAPI (blue). (A) Imaging. Note the increase in interstitial collagen levels in the mutant muscle compared to WT. (B) Quantification showed that Type I collagen levels were increased by 49% in the ∆Ig3-MuSK soleus (577.6 ± 33.7, n=47 and 860.6 ± 37.3, n=45 in WT and ∆Ig3-MuSK respectively, ****p< 0.0001, unpaired t-test; n=6 muscles per genotype; 5-6 sections/muscle. Supplementary Fig. S5. Supporting data for Western Blot in Fig. 7A (Soleus) and 7B (TA) showing total protein staining (Ponceau). Supplementary Fig. S6. P-S6 and p4EBP1 are down-regulated in ∆Ig3-MuSK soleus. Homogenates of 3-month-old soleus muscle from a different cohort of mice than used in Fig. 7 (see “Methods”) were separated by SDS-PAGE and probed with the indicated antibodies to phosphorylated (‘p’; A) or unphosphorylated (B) 4EBP1, S6, or Akt. Both blots were probed for GAPDH as a loading control. Total protein ( [file 13395_2023_329_MOESM1_ESM.zip › Supp Fig. S1 REVISE FINAL 9-16-2023.pdf]

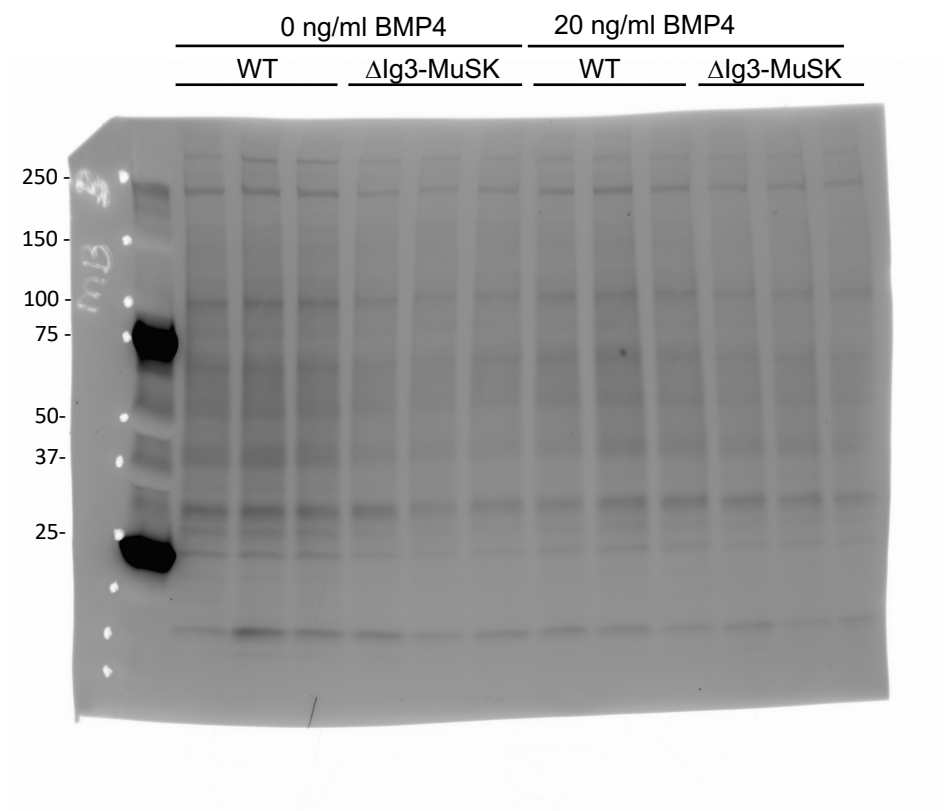

**Supplementary Figure S2.**

Supplement: Supplementary file 1 — Additional file 1: Supplementary Fig. S1. Immortalized WT or ∆Ig3-MuSK myoblasts were cultured in differentiating conditions for 3 days. The cultures were labeled with the pan-skeletal muscle myosin anti-MyHC (green; see “Methods”) and DAPI as described in methods. Note that ∆Ig3-MuSK cells differentiated into multi-nucleated myotubes that expressed MyHC. Supplementary Fig. S2. Supporting data for Western Blot in Fig. 2D showing total protein visualized using the No-Stain reagent (see “Methods”). Supplementary Fig. S3. Agrin-induced AChR clustering is comparable in WT and ∆Ig3-MuSK myotubes. WT and ∆Ig3-MuSK cultured primary myotubes were treated with agrin for 16 hr. (A) Visualization of AChR distribution. AChR clusters are denoted by arrows. (B) Quantification of AChR clusters. The agrin response was comparable in WT and ∆Ig3-MuSK myotubes (two-way ANOVA with Bonferroni’s multiple comparisons). Supplementary Fig. S4. Increased type I collagen levels in ∆Ig3-MuSK soleus. Sections of 3-month-old soleus muscle from WT and ∆Ig3-MuSK were stained with antibodies to Type I Collagen (red) and DAPI (blue). (A) Imaging. Note the increase in interstitial collagen levels in the mutant muscle compared to WT. (B) Quantification showed that Type I collagen levels were increased by 49% in the ∆Ig3-MuSK soleus (577.6 ± 33.7, n=47 and 860.6 ± 37.3, n=45 in WT and ∆Ig3-MuSK respectively, ****p< 0.0001, unpaired t-test; n=6 muscles per genotype; 5-6 sections/muscle. Supplementary Fig. S5. Supporting data for Western Blot in Fig. 7A (Soleus) and 7B (TA) showing total protein staining (Ponceau). Supplementary Fig. S6. P-S6 and p4EBP1 are down-regulated in ∆Ig3-MuSK soleus. Homogenates of 3-month-old soleus muscle from a different cohort of mice than used in Fig. 7 (see “Methods”) were separated by SDS-PAGE and probed with the indicated antibodies to phosphorylated (‘p’; A) or unphosphorylated (B) 4EBP1, S6, or Akt. Both blots were probed for GAPDH as a loading control. Total protein ( [file 13395_2023_329_MOESM1_ESM.zip › Supp Fig. S2 REVISE FINAL 9-16-2023.pdf]

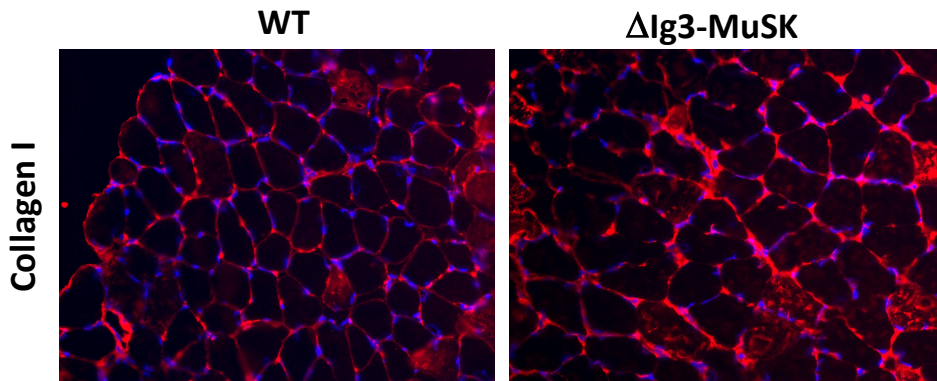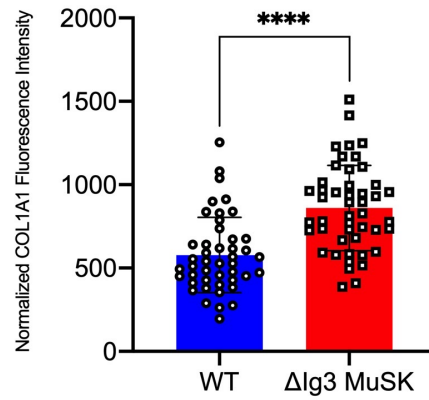

Supplementary Figure S4.

Supplement: Supplementary file 1 — Additional file 1: Supplementary Fig. S1. Immortalized WT or ∆Ig3-MuSK myoblasts were cultured in differentiating conditions for 3 days. The cultures were labeled with the pan-skeletal muscle myosin anti-MyHC (green; see “Methods”) and DAPI as described in methods. Note that ∆Ig3-MuSK cells differentiated into multi-nucleated myotubes that expressed MyHC. Supplementary Fig. S2. Supporting data for Western Blot in Fig. 2D showing total protein visualized using the No-Stain reagent (see “Methods”). Supplementary Fig. S3. Agrin-induced AChR clustering is comparable in WT and ∆Ig3-MuSK myotubes. WT and ∆Ig3-MuSK cultured primary myotubes were treated with agrin for 16 hr. (A) Visualization of AChR distribution. AChR clusters are denoted by arrows. (B) Quantification of AChR clusters. The agrin response was comparable in WT and ∆Ig3-MuSK myotubes (two-way ANOVA with Bonferroni’s multiple comparisons). Supplementary Fig. S4. Increased type I collagen levels in ∆Ig3-MuSK soleus. Sections of 3-month-old soleus muscle from WT and ∆Ig3-MuSK were stained with antibodies to Type I Collagen (red) and DAPI (blue). (A) Imaging. Note the increase in interstitial collagen levels in the mutant muscle compared to WT. (B) Quantification showed that Type I collagen levels were increased by 49% in the ∆Ig3-MuSK soleus (577.6 ± 33.7, n=47 and 860.6 ± 37.3, n=45 in WT and ∆Ig3-MuSK respectively, ****p< 0.0001, unpaired t-test; n=6 muscles per genotype; 5-6 sections/muscle. Supplementary Fig. S5. Supporting data for Western Blot in Fig. 7A (Soleus) and 7B (TA) showing total protein staining (Ponceau). Supplementary Fig. S6. P-S6 and p4EBP1 are down-regulated in ∆Ig3-MuSK soleus. Homogenates of 3-month-old soleus muscle from a different cohort of mice than used in Fig. 7 (see “Methods”) were separated by SDS-PAGE and probed with the indicated antibodies to phosphorylated (‘p’; A) or unphosphorylated (B) 4EBP1, S6, or Akt. Both blots were probed for GAPDH as a loading control. Total protein ( [file 13395_2023_329_MOESM1_ESM.zip › Supp Fig. S4 REVISE FINAL 9-16-23-2023.pdf]

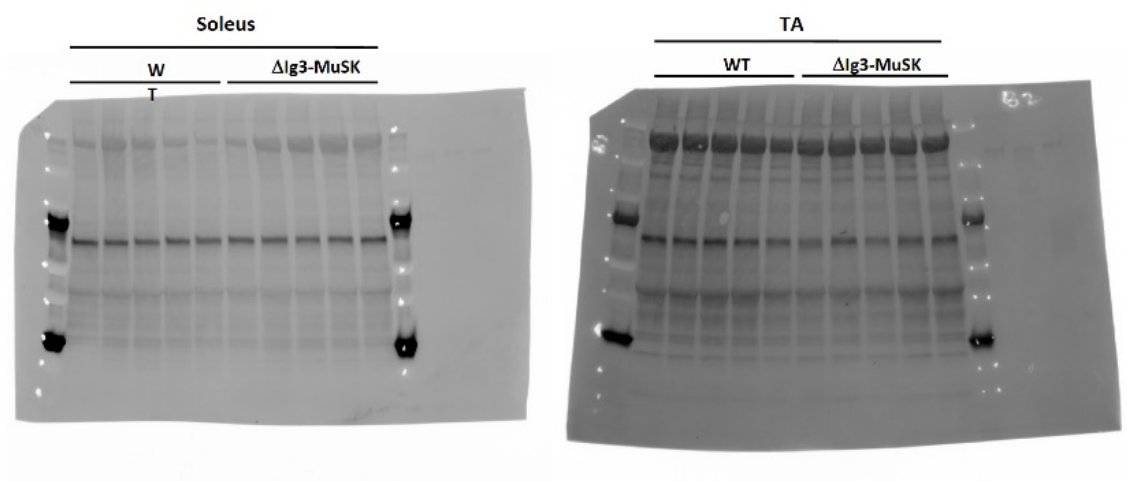

**Supplementary Figure S5.**

Supplement: Supplementary file 1 — Additional file 1: Supplementary Fig. S1. Immortalized WT or ∆Ig3-MuSK myoblasts were cultured in differentiating conditions for 3 days. The cultures were labeled with the pan-skeletal muscle myosin anti-MyHC (green; see “Methods”) and DAPI as described in methods. Note that ∆Ig3-MuSK cells differentiated into multi-nucleated myotubes that expressed MyHC. Supplementary Fig. S2. Supporting data for Western Blot in Fig. 2D showing total protein visualized using the No-Stain reagent (see “Methods”). Supplementary Fig. S3. Agrin-induced AChR clustering is comparable in WT and ∆Ig3-MuSK myotubes. WT and ∆Ig3-MuSK cultured primary myotubes were treated with agrin for 16 hr. (A) Visualization of AChR distribution. AChR clusters are denoted by arrows. (B) Quantification of AChR clusters. The agrin response was comparable in WT and ∆Ig3-MuSK myotubes (two-way ANOVA with Bonferroni’s multiple comparisons). Supplementary Fig. S4. Increased type I collagen levels in ∆Ig3-MuSK soleus. Sections of 3-month-old soleus muscle from WT and ∆Ig3-MuSK were stained with antibodies to Type I Collagen (red) and DAPI (blue). (A) Imaging. Note the increase in interstitial collagen levels in the mutant muscle compared to WT. (B) Quantification showed that Type I collagen levels were increased by 49% in the ∆Ig3-MuSK soleus (577.6 ± 33.7, n=47 and 860.6 ± 37.3, n=45 in WT and ∆Ig3-MuSK respectively, ****p< 0.0001, unpaired t-test; n=6 muscles per genotype; 5-6 sections/muscle. Supplementary Fig. S5. Supporting data for Western Blot in Fig. 7A (Soleus) and 7B (TA) showing total protein staining (Ponceau). Supplementary Fig. S6. P-S6 and p4EBP1 are down-regulated in ∆Ig3-MuSK soleus. Homogenates of 3-month-old soleus muscle from a different cohort of mice than used in Fig. 7 (see “Methods”) were separated by SDS-PAGE and probed with the indicated antibodies to phosphorylated (‘p’; A) or unphosphorylated (B) 4EBP1, S6, or Akt. Both blots were probed for GAPDH as a loading control. Total protein ( [file 13395_2023_329_MOESM1_ESM.zip › Supp Fig. S5 Final REVISE 9-16-2023.pdf]

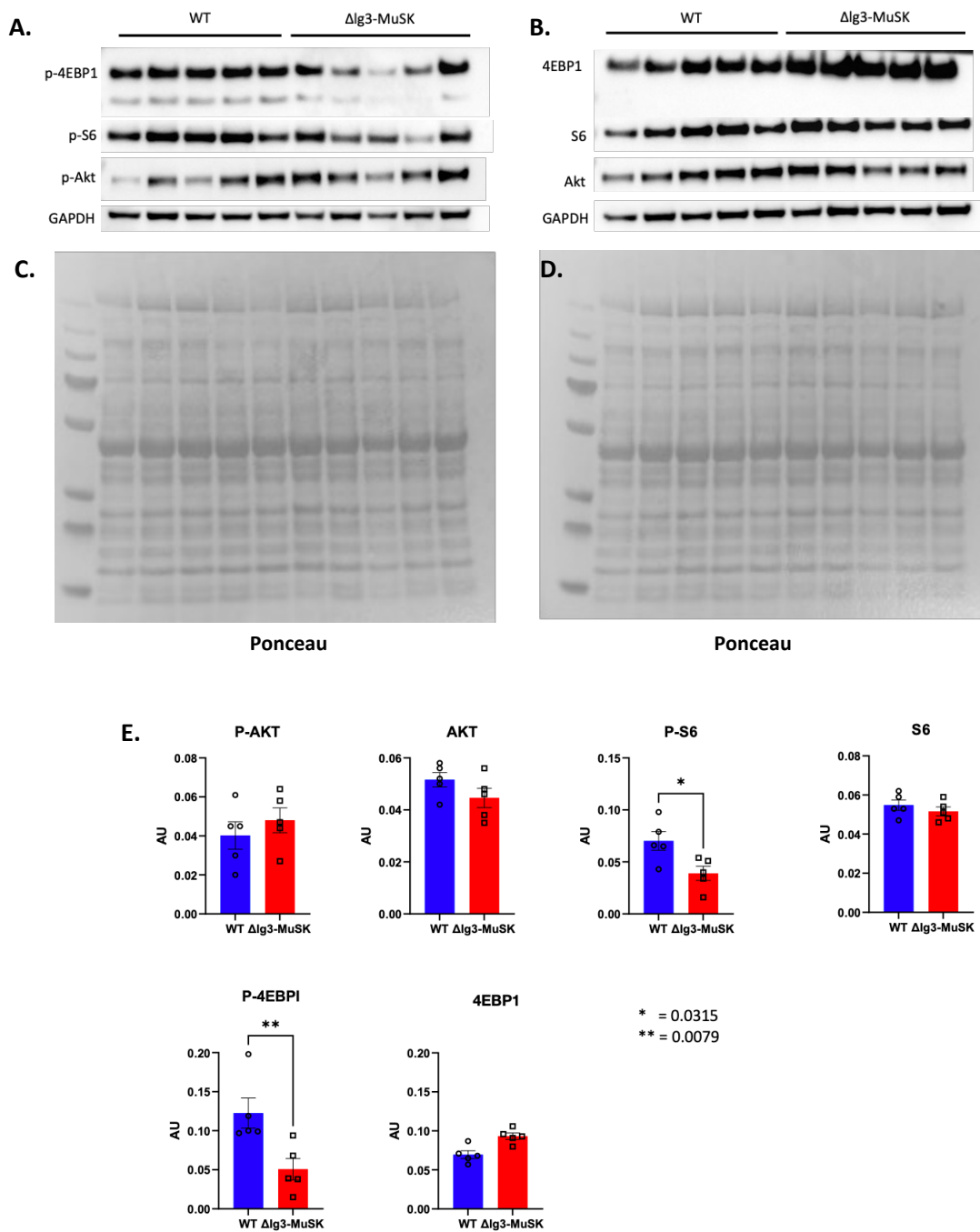

Supplementary Figure S6.

Supplement: Supplementary file 1 — Additional file 1: Supplementary Fig. S1. Immortalized WT or ∆Ig3-MuSK myoblasts were cultured in differentiating conditions for 3 days. The cultures were labeled with the pan-skeletal muscle myosin anti-MyHC (green; see “Methods”) and DAPI as described in methods. Note that ∆Ig3-MuSK cells differentiated into multi-nucleated myotubes that expressed MyHC. Supplementary Fig. S2. Supporting data for Western Blot in Fig. 2D showing total protein visualized using the No-Stain reagent (see “Methods”). Supplementary Fig. S3. Agrin-induced AChR clustering is comparable in WT and ∆Ig3-MuSK myotubes. WT and ∆Ig3-MuSK cultured primary myotubes were treated with agrin for 16 hr. (A) Visualization of AChR distribution. AChR clusters are denoted by arrows. (B) Quantification of AChR clusters. The agrin response was comparable in WT and ∆Ig3-MuSK myotubes (two-way ANOVA with Bonferroni’s multiple comparisons). Supplementary Fig. S4. Increased type I collagen levels in ∆Ig3-MuSK soleus. Sections of 3-month-old soleus muscle from WT and ∆Ig3-MuSK were stained with antibodies to Type I Collagen (red) and DAPI (blue). (A) Imaging. Note the increase in interstitial collagen levels in the mutant muscle compared to WT. (B) Quantification showed that Type I collagen levels were increased by 49% in the ∆Ig3-MuSK soleus (577.6 ± 33.7, n=47 and 860.6 ± 37.3, n=45 in WT and ∆Ig3-MuSK respectively, ****p< 0.0001, unpaired t-test; n=6 muscles per genotype; 5-6 sections/muscle. Supplementary Fig. S5. Supporting data for Western Blot in Fig. 7A (Soleus) and 7B (TA) showing total protein staining (Ponceau). Supplementary Fig. S6. P-S6 and p4EBP1 are down-regulated in ∆Ig3-MuSK soleus. Homogenates of 3-month-old soleus muscle from a different cohort of mice than used in Fig. 7 (see “Methods”) were separated by SDS-PAGE and probed with the indicated antibodies to phosphorylated (‘p’; A) or unphosphorylated (B) 4EBP1, S6, or Akt. Both blots were probed for GAPDH as a loading control. Total protein ( [file 13395_2023_329_MOESM1_ESM.zip › Supp Fig. S6 REVISE FINAL 9-16-2023.pdf]

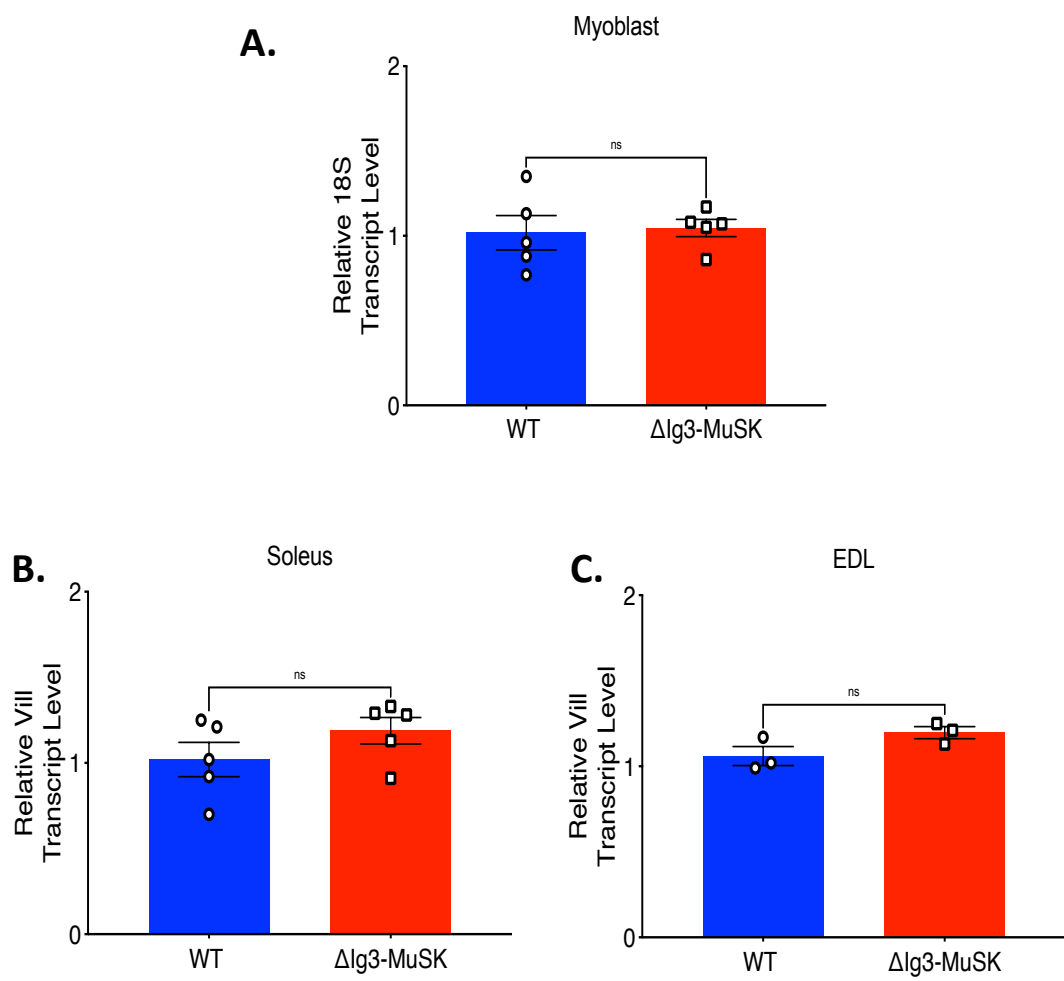

**Supplementary Figure S7.**

Supplement: Supplementary file 1 — Additional file 1: Supplementary Fig. S1. Immortalized WT or ∆Ig3-MuSK myoblasts were cultured in differentiating conditions for 3 days. The cultures were labeled with the pan-skeletal muscle myosin anti-MyHC (green; see “Methods”) and DAPI as described in methods. Note that ∆Ig3-MuSK cells differentiated into multi-nucleated myotubes that expressed MyHC. Supplementary Fig. S2. Supporting data for Western Blot in Fig. 2D showing total protein visualized using the No-Stain reagent (see “Methods”). Supplementary Fig. S3. Agrin-induced AChR clustering is comparable in WT and ∆Ig3-MuSK myotubes. WT and ∆Ig3-MuSK cultured primary myotubes were treated with agrin for 16 hr. (A) Visualization of AChR distribution. AChR clusters are denoted by arrows. (B) Quantification of AChR clusters. The agrin response was comparable in WT and ∆Ig3-MuSK myotubes (two-way ANOVA with Bonferroni’s multiple comparisons). Supplementary Fig. S4. Increased type I collagen levels in ∆Ig3-MuSK soleus. Sections of 3-month-old soleus muscle from WT and ∆Ig3-MuSK were stained with antibodies to Type I Collagen (red) and DAPI (blue). (A) Imaging. Note the increase in interstitial collagen levels in the mutant muscle compared to WT. (B) Quantification showed that Type I collagen levels were increased by 49% in the ∆Ig3-MuSK soleus (577.6 ± 33.7, n=47 and 860.6 ± 37.3, n=45 in WT and ∆Ig3-MuSK respectively, ****p< 0.0001, unpaired t-test; n=6 muscles per genotype; 5-6 sections/muscle. Supplementary Fig. S5. Supporting data for Western Blot in Fig. 7A (Soleus) and 7B (TA) showing total protein staining (Ponceau). Supplementary Fig. S6. P-S6 and p4EBP1 are down-regulated in ∆Ig3-MuSK soleus. Homogenates of 3-month-old soleus muscle from a different cohort of mice than used in Fig. 7 (see “Methods”) were separated by SDS-PAGE and probed with the indicated antibodies to phosphorylated (‘p’; A) or unphosphorylated (B) 4EBP1, S6, or Akt. Both blots were probed for GAPDH as a loading control. Total protein ( [file 13395_2023_329_MOESM1_ESM.zip › Supp Fig. S7 REVISE FINAL 9-16-2023.pdf]
