# Supplementary material for: The MuSK-BMP pathway maintains myofiber size in slow muscle through regulation of Akt-mTOR signaling
Source: Skelet Muscle. 2024 Jan 3;14:1. doi: 10.1186/s13395-023-00329-9 (PMC10763067; doi:10.1186/s13395-023-00329-9)
Supplement: Supplementary file 2 — Additional file 2: Table S1. Differentially-expressed genes in WT and ∆Ig3-MuSK TA. Table S2. Differentially-expressed genes in WT and ∆Ig3-MuSK Soleus. Table S3. GO terms for ∆Ig3-MuSK soleus and TA. Table S4. Dysregulated Akt-mTOR pathway genes in the soleus compared to TA. Table S5. Atrogenes expression in WT and ∆Ig3-MuSK soleus and TA muscle. [file 13395_2023_329_MOESM2_ESM.zip › Table S4 REVISED 8-2023 SkM.docx]

**Supplemental Table S4**

**IGF1-Akt-mTOR genes selectively dysregulated in ΔIg3-MuSK soleus**

|  | **SOL** | **TA** |
| --- | --- | --- |
| ***Gene*** | ***Adj p-value*** | ***Adj p-value*** |
| **Igf2bp2** | **0.015** | NA |
| **Igfbp2** | **0.031** | NA |
| **Igf1** | **0.032** | 0.32 |
| **Txnip** | **0.043** | 0.31 |
| Gm15441^*^ | 0.051 | 0.59 |
| Irs2 | 0.055 | 0.12 |
| Rhebl1 | 0.060 | NA |
| Irs1 | 0.064 | 0.38 |

*lncRNA antisense to Txnip

**Bold: p <0.05**
